# Optimization of Polycaprolactone and Type I Collagen Scaffold for Tendon Tissue Regeneration

**DOI:** 10.7759/cureus.56930

**Published:** 2024-03-25

**Authors:** Craig Cady, Kalyani Nair, Hugo C Rodriguez, Brandon Rust, Samir Ghandour, Anish Potty, Ashim Gupta

**Affiliations:** 1 Biology, Bradley University, Peoria, USA; 2 Mechanical Engineering, Bradley University, Peoria, USA; 3 Orthopedic Surgery, Larkin Community Hospital, Miami, USA; 4 Medicine, Nova Southeastern University Dr. Kiran C. Patel College of Osteopathic Medicine, Fort Lauderdale, USA; 5 Radiology, Harvard Medical School, Boston, USA; 6 Orthopedics, South Texas Orthopaedic Research Institute, Laredo, USA; 7 Regenerative Medicine, Future Biologics, Lawrenceville, USA; 8 Orthopedics and Regenerative Medicine, Regenerative Orthopedics, Noida, IND; 9 Regenerative Medicine, BioIntegrate, Lawrenceville, USA

**Keywords:** tendons, collagen, polycaprolactone, electrospinning, biomaterials, scaffolds, regenerative medicine, tissue engineering, musculoskeletal injuries

## Abstract

Introduction

Collagen synthesis is vital for restoring musculoskeletal tissues, particularly in tendon and ligamentous structures. Tissue engineering utilizes scaffolds for cell adhesion and differentiation. Although synthetic scaffolds offer initial strength, their long-term stability is surpassed by biological scaffolds. Combining polycaprolactone (PCL) toughness with collagen in scaffold design, this study refines fabrication via electrospinning, aiming to deliver enduring biomimetic matrices for widespread applications in musculoskeletal repair.

Methods

Electrospinning employed four solutions with varied collagen and PCL concentrations, dissolved in chloroform, methanol, and hexafluoro-2-propanol. Solutions were combined to yield 60 mg/mL concentrations with different collagen/PCL ratios. Electrospinning at 12-14kV voltage produced scaffolds, followed by vacuum-drying. Collagen coating was applied to PCL and 15% collagen/PCL scaffolds using a 0.1% collagen solution. SEM characterized fiber morphology, tensile testing was conducted to determine the mechanical properties of the scaffold, and Fourier-transform infrared (FTIR) spectroscopy analyzed scaffold composition. Atomic force microscopy (AFM) analyzed the stiffness properties of individual fibers, and a finite element model was developed to predict the mechanical properties. Cell culture involved seeding human bone marrow mesenchymal stem cells onto scaffolds, which were assessed through Alamar Blue assay and confocal imaging.

Results

Various scaffolds (100% PCL, PCL-15% collagen, PCL-25% collagen, PCL-35% collagen) were fabricated to emulate the extracellular matrix, revealing collagen's impact on fiber diameter reduction with increasing concentration. Tensile testing highlighted collagen's initial enhancement of mechanical strength, followed by a decline beyond PCL-15% collagen. FTIR spectroscopy detected potential hydrogen bonding between collagen and PCL. A finite element model predicted scaffold response to external forces which was validated by the tensile test data. Cell viability and proliferation assays demonstrated successful plating on all scaffolds, with optimal proliferation observed in PCL-25% collagen. Confocal imaging confirmed stem cell integration into the three-dimensional material. Collagen coating preserved nanofiber morphology, with no significant changes in diameter. Coating of collagen significantly altered the tensile strength of the scaffolds at the macro scale. AFM highlighted stiffness differences between PCL and collagen-coated PCL mats at the single fiber scale. The coating process did not significantly enhance initial cell attachment but promoted increased proliferation on collagen-coated PCL scaffolds.

Conclusion

The study reveals collagen-induced mechanical and morphological alterations, influencing fiber alignment, diameter, and chemical composition while emphasizing scaffolds' vital role in providing a controlled niche for stem cell proliferation and differentiation. The optimization of each of these scaffold characteristics and subsequent finite element modeling can lead to highly repeatable and ideal scaffold properties for stem cell integration and proliferation.

## Introduction

The restoration of musculoskeletal tissue damage to bones, tendons, and ligaments heavily relies on the proficiency of collagen synthesis [1]. ​​Conventional management of tendon injuries involves mobilization therapy, anti-inflammatory medications, and surgical interventions, prioritizing symptomatic relief over definitive structural repair [2]. Tendons, constituting up to 80% of the total extracellular matrix protein, prominently feature type I collagen, which imparts tensile strength to connective tissues, acting as a primary structural framework [2-4]. In the context of orthopedic surgery, strategically enhancing the structural integrity of such tissue through regenerative therapeutics emerged as a crucial approach to restoring musculoskeletal function and mitigating long-term degeneration.

Regenerative medicine employs biological alternatives or tissue constructs to restore, maintain, or enhance function in response to physiological, pathological, or mechanical conditions or trauma [5]. Biomaterials, including scaffolds, are frequently utilized for the repair of various anatomical defects. Often composed of differing materials, scaffolds serve as a media or framework that provides an environment for stem cells or other cells to adhere, proliferate, and differentiate into the desired tissue [5]. While traditional synthetic scaffolds provide initial strength and stability, they lack long-term stability and biocompatibility which may undermine their applications and intended outcomes. On the other hand, biological scaffolds derived from human or animal connective tissue circumvent this by exhibiting natural permeability and enhanced biological performance in vivo [6].

Biopolymers play a crucial role in scaffold construction, with polycaprolactone (PCL) standing out as a noteworthy material [5]. PCL, characterized by its aliphatic and semi-crystalline nature, demonstrates exceptional toughness, mechanical strength, and biocompatibility [5,7]. Its two-year degradation rate not only reduces local acidification and inflammation but also extends its applicability in tissue engineering [5]. In synergy with PCL, collagen significantly enhances scaffold properties that support cell growth, owing to its bio-inductive, mechanically compatible, and biodegradable characteristics. Numerous studies have provided evidence of collagen's efficacy in improving cell adhesion, stimulating bone cell proliferation, and enhancing osteogenic cell differentiation, particularly in facilitating cellular development and handling efficiency during implantation [5].

The combination of PCL and collagen in biological scaffold design leverages their respective benefits, and the process of electrospinning is employed to fabricate these scaffolds. Electrospinning yields fibers possessing favorable attributes, including elevated porosity, expansive surface area, and continuous lengths, facilitating precise control over fiber alignment and morphology [5]. However, recent literature highlights the process's mechanical shortcomings due to limited cell infiltration within the scaffold [8,9]. Therefore, this study aims to fabricate an optimized biological scaffold consisting of an adequate amount of type I collagen in PCL to provide sufficient strength and cell growth potential for tendon regeneration. We hypothesize that variable collagen concentrations incorporated into the scaffold biopolymer combination would alter the mechanical and biocompatible properties of the scaffold. Additionally, to predict the macro-scale properties of the biological scaffold, we constructed a model using the fabricated nanofibers optimized for reproducible integration within native tendon tissue architecture.

## Materials and methods

Electrospinning

Four solutions with varying concentrations of type I collagen (Sigma, St. Louis, USA) and PCL (Sigma, St. Louis, USA) were utilized in this study. PCL was dissolved separately in 75% chloroform (Sigma, St. Louis, USA) and 25% methanol (Sigma, St. Louis, USA), and type I collagen was dissolved in 1,1,1,3,3,3-hexafluoro-2-propanol (Sigma, St. Louis, USA). The separately dissolved solutions were then combined drop-wise and allowed to mix on a magnetic stir plate for 24 hours. The final combined solution had a concentration of 60 mg/mL and contained either 0, 9, 15, or 21 mg of collagen corresponding to the 100% PCL and 15%, 25%, and 35% collagen/PCL scaffolds, respectively. In the solution that contained no collagen, 1,1,1,3,3,3-hexafluoro-2-propanol was still added and allowed to mix for 24 hours to ensure that the solvent dynamics during the electrospinning process remained constant.

The aforementioned solutions were drawn into a 20 mL glass syringe with an 18-gauge needle and placed onto a syringe pump (Thermo Fisher Scientific, Waltham, USA). A voltage of 12-14 kV was applied via a Gamma HV ES30P-5W/DAM power supply (Thermo Fisher Scientific, Waltham, USA) to the tip of the syringe needle and grounded at a copper collecting plate. The collecting plate was located 13 cm from the tip of the syringe needle and the syringe pump ejected the solution at a controlled rate of 2.0 ml/hr. The solution was electrospun for 2.5 hours and the resulting scaffolds were dried under vacuum for 2.5 hours to remove any remaining solvents that may have been present in the scaffold.

Collagen coating

After the electrospinning process was complete, PCL and 15% collagen/PCL scaffolds were coated with collagen. This process was done utilizing type I collagen dissolved in dimethyl sulfoxide (DMSO) (Sigma, St. Louis, USA). This solution was then further diluted utilizing phosphate-buffered saline (PBS) (Sigma, St. Louis, USA) until a final concentration of 0.1% collagen solution was obtained with minimal remaining DMSO. The collagen solution was then placed onto the electrospun scaffolds and allowed to incubate at 37°C for 24 hours. The excess solution was removed from the fibers, and the resulting scaffolds were cross-linked and sterilized using ultraviolet light.

Fiber characteristics and morphology

The PCL and PCL-collagen scaffolds were initially prepared for morphological characterization by cutting sections with an area of approximately 0.5 cm^2^ and attaching them to a JEOL (Japan Electron Optics Laboratory, Akishima, Japan) aluminum specimen mount with copper tape. These specimens were then sputter-coated with gold and palladium to prevent charging of the material. Fiber morphology of the PCL and PCL-collagen scaffolds were then observed utilizing a scanning electron microscopy (SEM) (JEOL 6060LV SEM, Peabody, USA) at an accelerating voltage of 15 kV and 15,000 times magnification. The diameter, orientation, and porosity of the individual fibers and scaffold were obtained from these SEM images and analyzed using ImageJ analysis software (National Institute of Health, Bethesda, USA).

Tensile testing

Specimens were cut out according to the American Society for Testing and Materials standard D-638-V. The thickness of these scaffolds was measured by a micrometer at four separate locations, with the average thickness from these measurements being utilized for subsequent calculations. The scaffolds were tested utilizing an Instron Universal testing machine model 4201 (Instron, Norwood, USA) with a 1kN load cell at 23°C and 50% humidity. The specimen for every scaffold had a gauge length of 7.62 mm and the crosshead speed was kept constant at 10 mm/min.

Fourier-transform infrared spectroscopy

Fourier-transform infrared (FTIR) spectroscopy was performed on a Bruker Tensor 37 FTIR Spectrometer (Brucker, Billerica, USA) at 74°C and 50% humidity to obtain absorbance spectra of all solutions and their resulting scaffolds. These spectra were then further examined to determine changes in absorbance amongst the various solutions and scaffolds.

Atomic force microscopy

Nanoindentation studies were performed on the PCL and PCL-collagen scaffolds at room temperature using Nanosurf C3000 atomic force microscopy (AFM) (Nanosurf AG, Liestal, Switzerland). A rectangular low-stress, silicon nitride (SiN) AFM tip with gold coating was utilized. Furthermore, the cantilever radius was less than 25 nm, with a frequency of 66 Hz and a spring constant of 0.284 N/m. The AFM was utilized in contact mode, and a topography map was obtained by recording defection when the cantilever moved along the scaffold. Additionally, force-deflection curves were obtained for each scaffold type. Images for each scaffold were obtained, and individual fibers from each image were utilized to determine the stiffness of the corresponding scaffold. Force tip-sample separation plots were obtained at 64 points along a line on each fiber. These plots were used to determine stiffness, maximum adhesive force, dissipation energy, and surface roughness for each type of mat. The force spectroscopy images were analyzed using SPIP Version 6.7.8 (Image Metrology, Lyngby, Denmark) and AtomicJ Version 1.8.2. SPIP software was also utilized to determine fiber diameter. Last, the surface roughness of each mat was analyzed using Nanosurf C3000 Version 3.8.6.4 software (Nanosurf, Liestal, Switzerland). The arithmetical mean roughness was determined with the following equation:

$Sa=1/n\sum_{i=1}^{n}\left | yi \right |$

where n is the number of points, y is the height (z) at a given pixel (i) in the image.

Modeling

A finite element model of each PCL/collagen scaffold was created using Abaqus (6.16-3 Student Edition, Simulia Corp, Providence, Rhode Island). SEM and AFM images were used to determine the geometric characteristics and relative positions of the nanofibers composing the mat. The diameters, lengths, orientation angles, and elastic moduli of selected fibers were all quantified. The material properties derived from the AFM analysis were used for the individual fibers. These characteristics were then utilized to create B33 (2-node cubic elements in space) elements and mesh optimization was performed.

Cell culture and cell plating

Adherent human bone marrow mesenchymal stem cells (hBMSCs) (provided by Dr. Darwin Prockop, Center for Gene Therapy, Tulane University Health Sciences Center, New Orleans, USA) were characterized by flow cytometry for immuno-positive expression of CD73, CD105, and negative expression of CD34, and the differentiation potential was confirmed for osteoblasts, chondrocytes, and adipocytes. Vials of hBMSCs cells were recovered from cryopreservation and seeded at a density of 5 x 10^5^ cells per T-75 cell culture treated flasks for expansion and cultured under 5% CO_2_ and ambient O_2_ at 37˚C for two days before plating onto scaffolds. Scaffolds were prepared by cutting 6.35 mm and 15.5 mm diameter discs and affixing them to 96 and 24 well-plates, respectively, with sterilized vacuum grease. The scaffolds were then hydrated for a minimum of two hours with Dulbecco’s modified eagle medium (DMEM) (Sigma, St. Louis, USA) at room temperature. After hydration, the cells were passaged with TrypLE Express (Thermo Fisher Scientific, Waltham, USA) and transferred at the appropriate volume and media to 24 and 96 well plates containing the scaffolds at a cell density of 1 x 10^4^. For PCL/collagen experiments the same seeding density was utilized on scaffolds containing 15%, 25%, and 35% collagen. The cells were allowed to plate for one hour prior to the addition of further media or proliferation assessment. After plating, the cells were allowed to proliferate for three and five days, respectively. Proliferation was assessed utilizing the Alamar Blue proliferation assay (Thermo Fisher, Waltham, USA) according to the manufacturer's suggested instructions. Cells were loaded with 5 µM of carboxyfluorescein diacetate (CFDA) fluorescein stain (Thermo Fisher, Waltham, USA) for confocal image analysis.

Confocal imaging

After proliferation, disks were fixed in cold 4% paraformaldehyde, rinsed with cold PBS, and mounted to large coverslips with Vectashield 4',6-diamidino-2-phenylindole (DAPI) mounting medium (Vector Laboratories, Burlingame, USA). These discs were then imaged with a Leica confocal microscope (Leica TCS SP5 spectral laser confocal microscope, Wetzlar, Germany) at fluorescein isothiocyanate and DAPI excitation wavelengths.

Statistical analysis

All measured variables for proliferation, fiber diameter, and max tensile strength were analyzed for significant differences utilizing a one-way ANOVA and Tukey post hoc test. AFM results were analyzed utilizing nonparametric independent tests and were performed using IBM SPSS Statistics for Windows, Version 24 (Released 2016; IBM Corp., Armonk, New York, USA) to compare the material properties of the PCL mats to the collagen-coated PCL mats. Statistical significance was set at p<0.05.

## Results

Effect of protein concentration during electrospinning on fiber morphology and diameter

**Figure 1:**
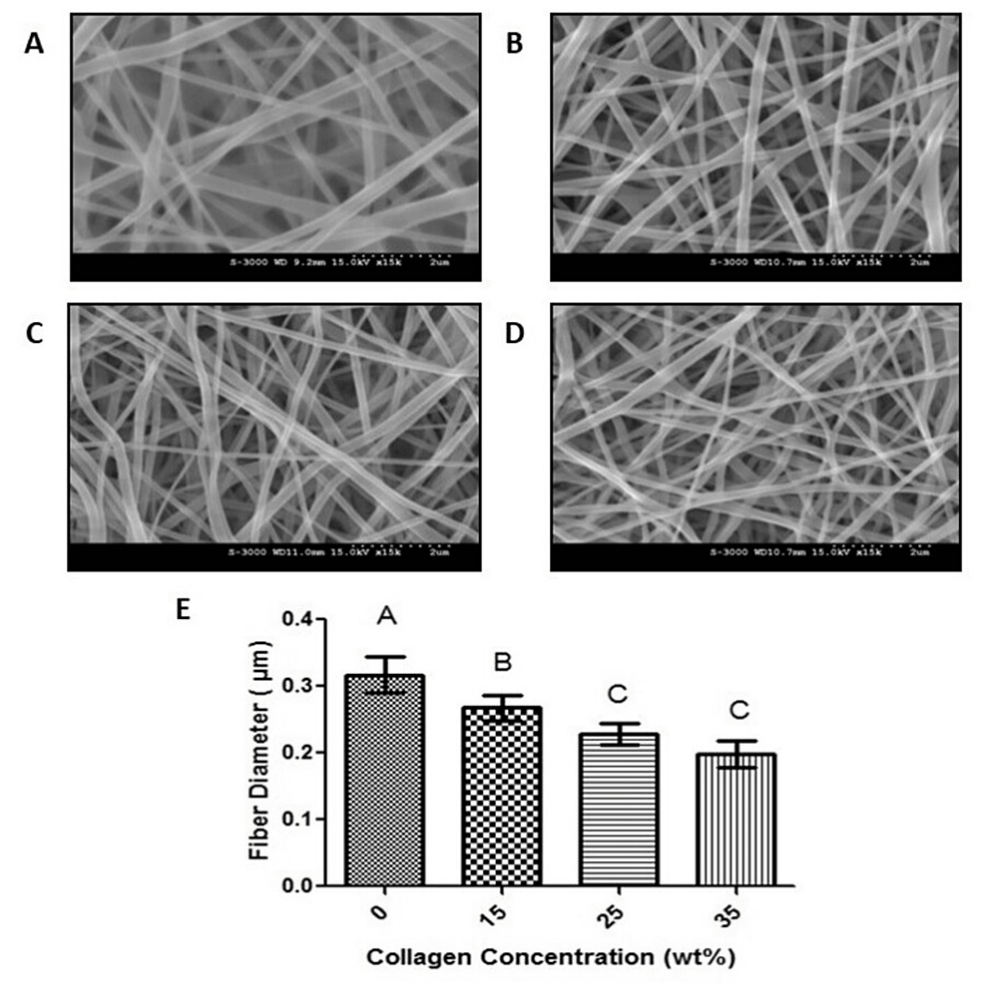
Scanning electron microscopy (SEM) images of (A) 100% polycaprolactone, (B) polycaprolactone-15% collagen, (C) polycaprolactone-25% collagen, (D) polycaprolactone-35% collagen; (E) ImageJ analysis indicating changes in fiber diameter with different collagen concentrations. Different letters above the error bars represent statistically significant differences between groups, similar letters were not significant, and error bars represent the standard error of the mean

The process of electrospinning was utilized to construct four distinct scaffolds with varying concentrations of PCL and collagen. Upon SEM analysis of these electrospun scaffolds, these scaffolds exhibited the morphologies shown in Figure 1. In each case, the scaffolds contained randomly aligned nanofibers which mimicked the extracellular matrix. Furthermore, each scaffold had minimal amounts of defects or beads present within the material. Each SEM image was further analyzed using ImageJ software to determine the effect of increasing amounts of electrospun collagen on fiber diameter (Figure 1). These results demonstrated that collagen when included in the process of electrospinning, acts to reduce the diameter of electrospun fibers as its overall concentration increases.

Mechanical and chemical characterization of PCL-collagen scaffolds

**Figure 2:**
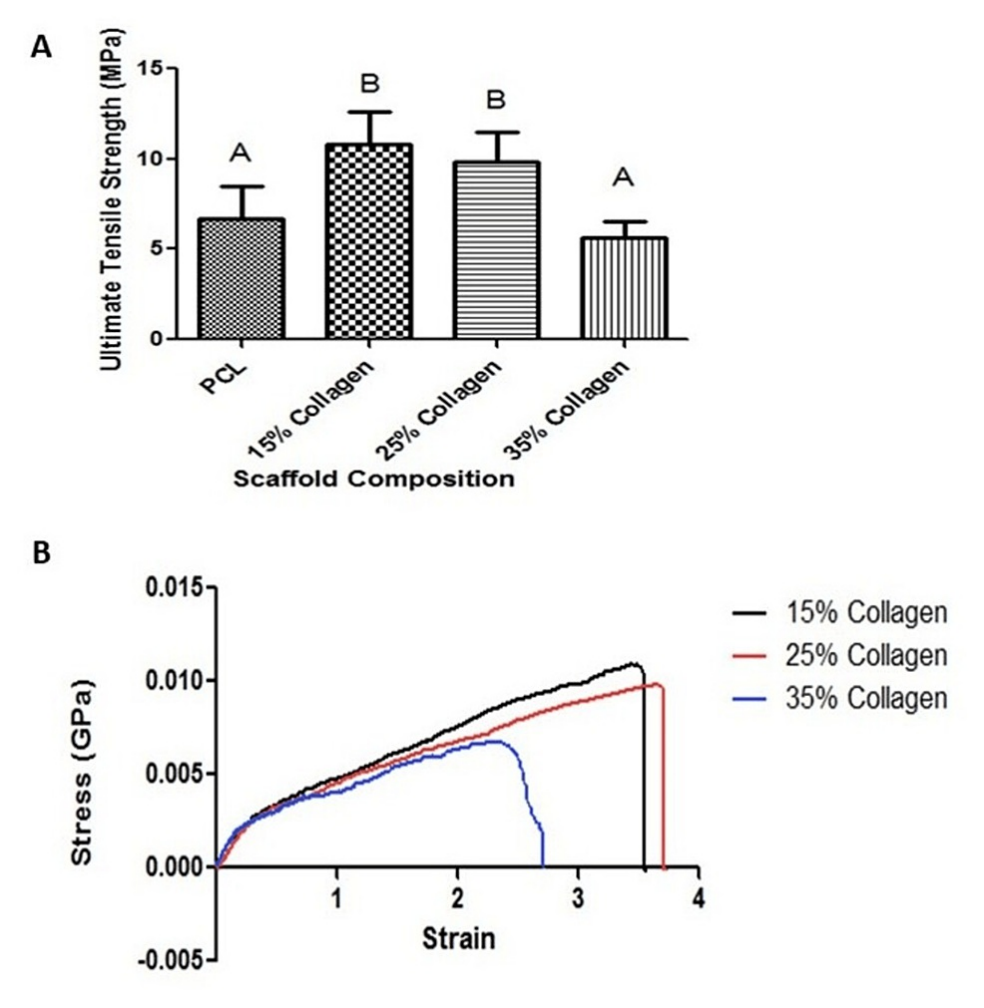
(A) ultimate tensile strength of electrospun scaffolds with various collagen concentrations, (B) stress vs strain curves for electrospun scaffolds with various amounts of collagen. Different letters above the error bars represent statistically significant differences between groups, similar letters were not significant, and error bars represent the standard error of the mean

To investigate the effect of PCL and collagen concentration on the mechanical properties of the constructed scaffolds, tensile testing was performed on multiple samples of each scaffold. Figure 2A displays the resulting ultimate tensile strength of each material. These values indicate that collagen concentration has a significant effect on the mechanical strength of the scaffold. Specifically, collagen acts to initially increase the ultimate tensile strength of the composite but ultimately decreases scaffold tensile strength when the concentration is increased beyond a certain percentage. Figure 2B shows the resulting stress-strain curves for various electrospun PCL-collagen scaffolds, demonstrating the change in mechanical properties of the scaffold with collagen concentration. Notably, the 35% collagen scaffold ultimately fails at a much lower percent elongation than the 15% or 25% collagen scaffolds.

**Figure 3:**
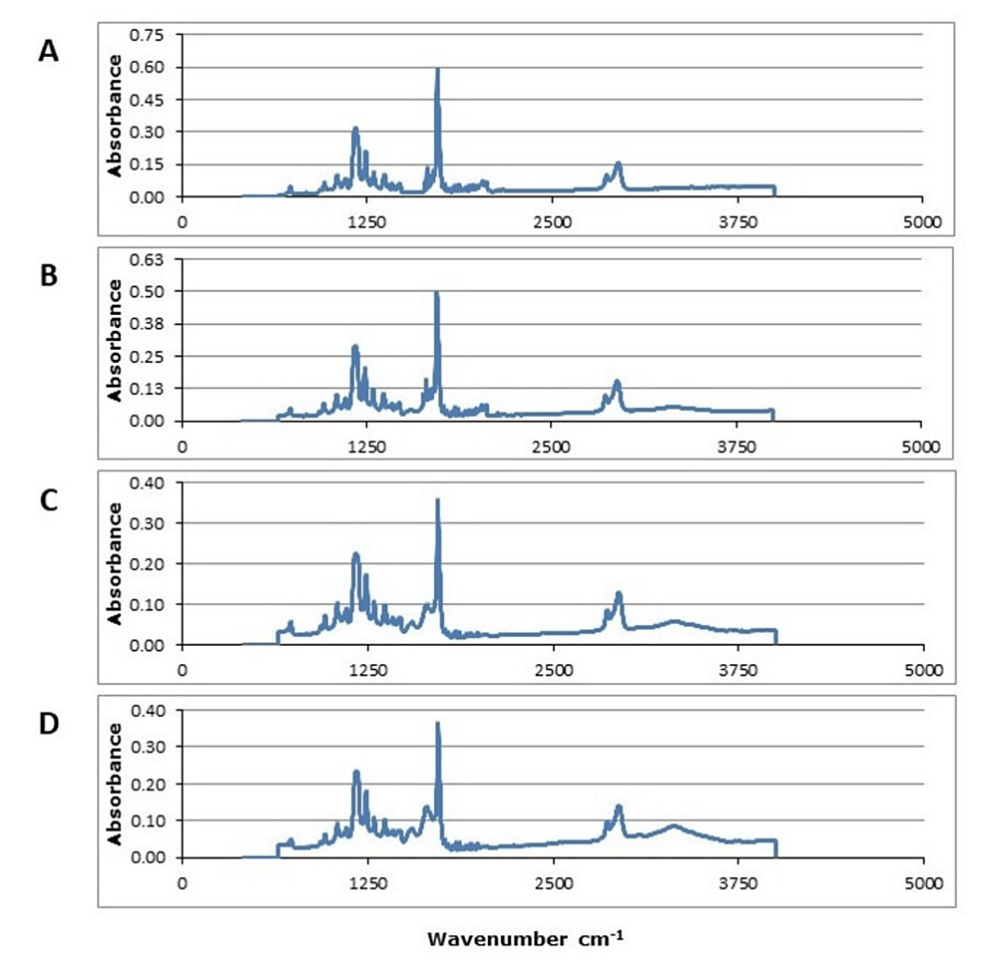
Fourier-transform infrared (FTIR) spectroscopy analysis of (A) polycaprolactone (PCL) scaffold, (B) PCL-15% collagen scaffold, (C) PCL-25% collagen scaffold, (D) PCL-35% collagen scaffold PCL: Polycaprolactone

Furthermore, FTIR analysis was performed on each of the scaffolds and the results are shown in Figure 3. As shown, there are changes in peaks at approximately 3300 cm^-1^ and 1725 cm^-1^. These peaks correspond to potential hydrogen bonding interactions between collagen and PCL as well as a delocalization of amide linkage lone pairs. However, none of the band changes in the FTIR analysis suggest that both collagen and PCL are fully integrated into the individual nanofibers that comprise the scaffold.

Modeling of scaffold dynamics

**Figure 4:**
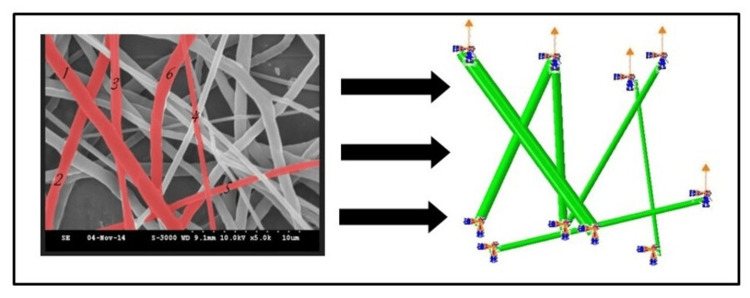
Abaqus was utilized to create a three-dimensional (3D) model that mimicked the morphology and properties of the nanofibers for a given scaffold

The various scaffolds were then analyzed utilizing AFM. The results of this analysis, along with the morphology given the SEM images, were utilized to construct the Abaqus model of the scaffolds as shown in Figure 4. This figure illustrates how various theoretical forces can be placed on the scaffold, and the resulting deformations to the scaffold can be determined.

Assessment of cell viability and proliferation on PCL-collagen scaffolds

**Figure 5:**
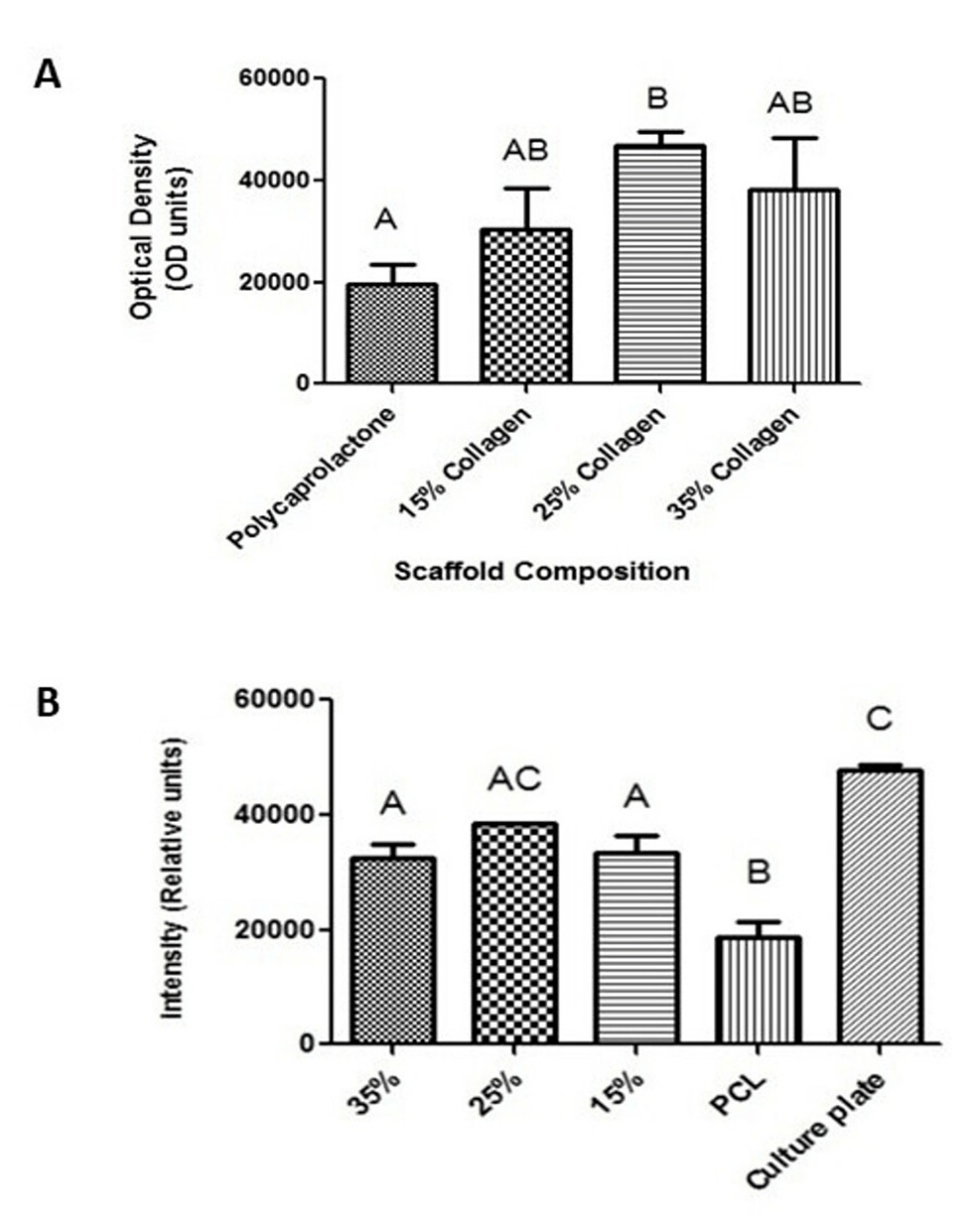
Human bone marrow mesenchymal stem cells (hBMSCs) were seeded onto various scaffolds and allowed to proliferate. (A) Alamar Blue performed after initial plating shows a relative number of stem cells adhered to the scaffold, (B) Alamar Blue performed after five days shows proliferation of stem cells on the various scaffolds. Different letters above the error bars represent statistically significant differences between groups, similar letters were not significant, n = 3 per experimental group, error bars represent standard error of the mean

After the completion of the physical and mechanical analysis of the scaffolds, each material was analyzed for its biocompatibility with adult stem cells. Cell proliferation assays were completed after the initial plating of hBMSCs to scaffolds containing increasing collagen concentrations (Figure 5A) and after five days in culture (Figure 5B). Initial plating proliferation results demonstrated cell plating was successful on all scaffolds while data after five days in culture demonstrated cell growth with only 25% collagen comparable to cell culture treated plate without scaffold.

**Figure 6:**
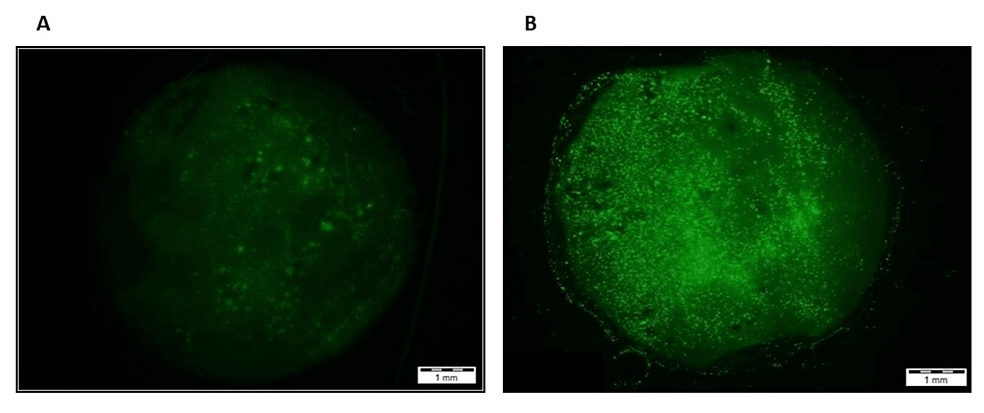
CFDA staining of hBMSCs seeded on: (A) PCL scaffold and (B) PCl-15% collagen scaffold (representative image) CFDA: Carboxyfluorescein diacetate; hBMSCs: Human bone marrow mesenchymal stem cells; PCL: Polycaprolactone

In addition to the proliferation studies, CFDA staining was performed on each constructed scaffold. The results, shown in Figure 6, indicate an increase in fluorescence on materials that included collagen.

**Figure 7:**
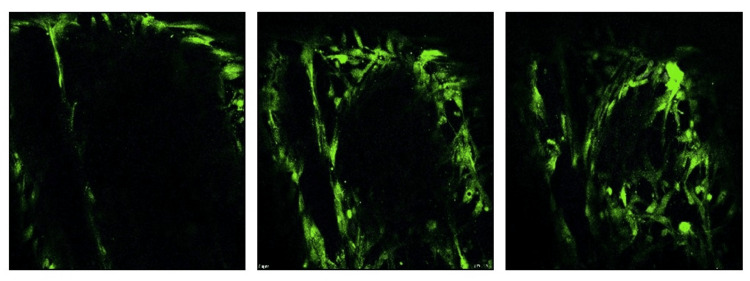
Confocal imaging of Z-stack images shows the integration of hBMSCs at multiple focal planes of a single PCL-collagen scaffold (representative images) hBMSCs: Human bone marrow mesenchymal stem cells; PCL: Polycaprolactone

Furthermore, confocal imaging was performed on scaffolds that had been seeded with stem cells. Figure 7 illustrates the presence of hBMSCs in multiple z stacks or focal planes, throughout the scaffold. This indicates that hBMSCs not only adhered to the scaffold but actively integrated into multiple layers of the three-dimensional material.

Effect of coating on fiber morphology, diameter, and mechanical properties

**Figure 8:**
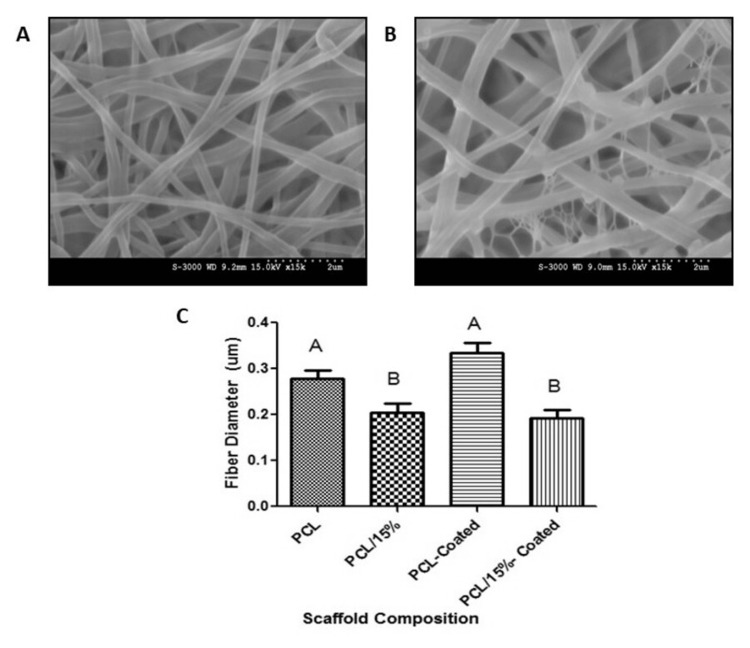
Scanning electron microscopy (SEM) images of (A) 100% polycaprolactone and collagen-coated, (B) polycaprolactone-15% collagen and collagen-coated, and (C) ImageJ analysis indicating changes in fiber diameter with collagen concentration. Different letters above the error bars represent statistically significant differences between groups, similar letters were not significant, and error bars represent the standard error of the mean

After initial analysis of the electrospun scaffolds, the PCL and PCL-15% collagen scaffolds were selected for further study. Each scaffold was further altered through a collagen coating process. Afterward, the scaffolds were again analyzed for changes in morphology as indicated in Figure 8. As shown, the morphology of each scaffold again mimicked the extracellular matrix, was randomly aligned in nature, and contained minimal amounts of beads or defects. Further analysis through ImageJ indicated that there was no significant difference in fiber diameter after the coating process. As such, the coating process did not cause significant and large alternations to the morphology of the electrospun scaffolds.

**Figure 9:**
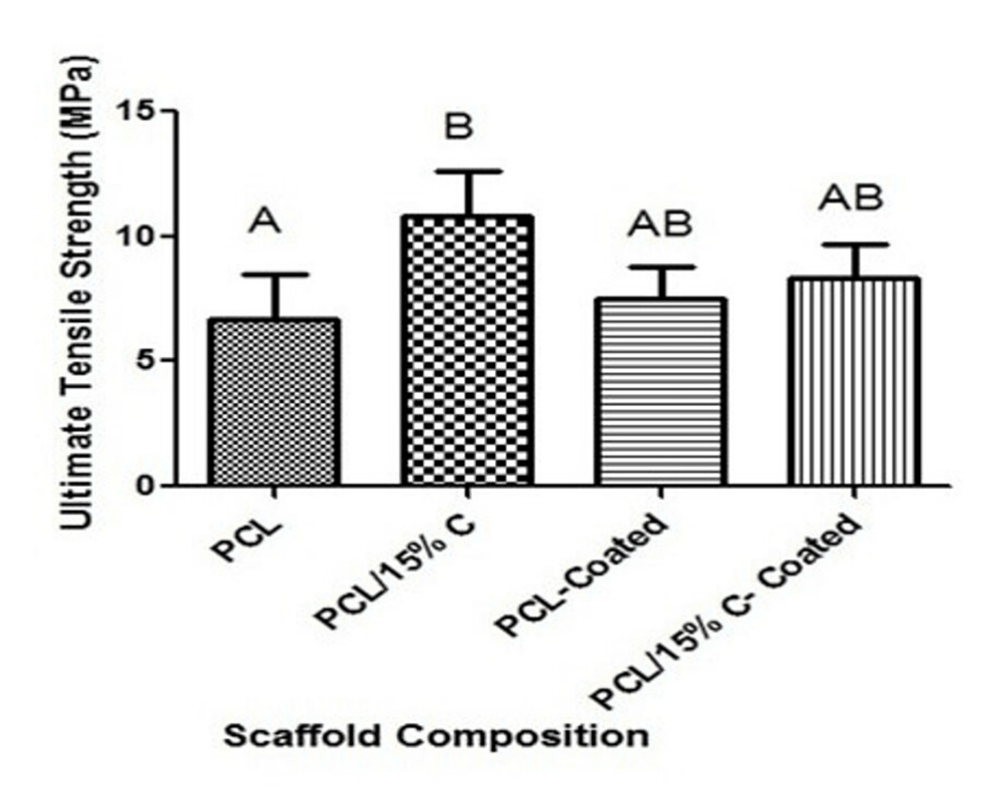
Ultimate tensile strength of various electrospun scaffolds with collagen and collagen coating. Different letters above the error bars represent statistically significant differences between groups, similar letters were not significant, and error bars represent the standard error of the mean

After morphological assessment of these coated nanofibers, the ultimate tensile strength of each scaffold was again determined utilizing tensile testing (Figure 9). As shown in the figure, it appeared that the coating process slightly increased the tensile strength of PCL but decreased the tensile strength of the PCL-15% collagen scaffold. In contrast, the PCL-15% collagen scaffold was qualitatively much more hydrophilic, which resulted in the coating solution being absorbed by this scaffold. The result was the trends shown in Figure 9, none of these mechanical property alterations due to the coating solution were significantly different.

**Figure 10:**
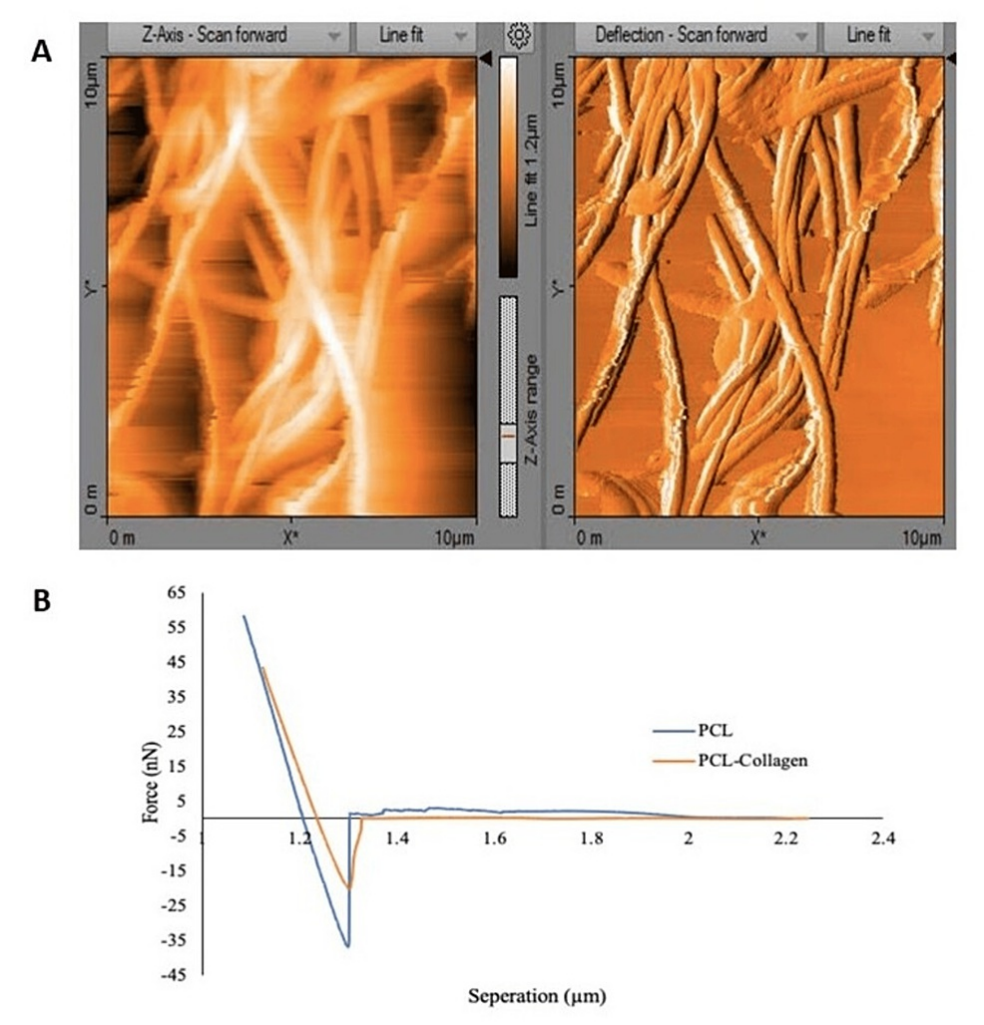
Atomic force microscopy analysis indicates: (A) surface image of the polycaprolactone (PCL) scaffold and (B) withdrawal curve comparison for PCL and PCL with collagen coating fibers PCL: Polycaprolactone

The PCL and collagen-coated PCL samples were then analyzed utilizing AFM. The surface and stiffness properties were quantified and compared, and images were taken for the PCL and PCL-collagen mats using the Nanosurf. Furthermore, the force tip-separation withdrawal curves were used to determine the fibers’ stiffness properties. Figure 10 illustrates sample Nanosurf images and withdrawal curves for these mats. A slight shift in the curve indicates the difference in stiffness properties of the fibers from the two scaffolds. It should be noted that the stiffness, maximum adhesion force, and distortion energy values were significantly higher in PCL mats than in collagen-coated PCL scaffolds (p<0.0001) (Table 1). In contrast, the mean surface roughness (p<0.0001) and diameter (p<0.001) values were higher for the PCL-collagen scaffolds (Table 1).

**Table 1:** Stiffness, diameter, maximum adhesion force, distortion energy and mean surface roughness of polycaprolactone (PCL) scaffold versus collagen-coated PCL scaffold PCL: Polycaprolactone

		Polycaprolactone (PCL)	Collagen-coated polycaprolactone	p-value
Stiffness (N/m)	Mean	0.501	0.436	<0.0001
	Standard deviation	0.0737	0.0471	
Diameter (nm)	Mean	499.41	522.39	<0.001
	Standard deviation	149.63	104.91	
Maximum adhesion force (nN)	Mean	34.54	17.90	<0.0001
	Standard deviation	17.29	6.82	
Distortion energy (fJ)	Mean	2.59	1.00	<0.0001
	Standard deviation	2.13	0.545	
Mean surface roughness (nm)	Mean	96.48	272.23	<0.0001
	Standard deviation	28.35	180.22	

Effects of collagen coating on cellular proliferation

**Figure 11:**
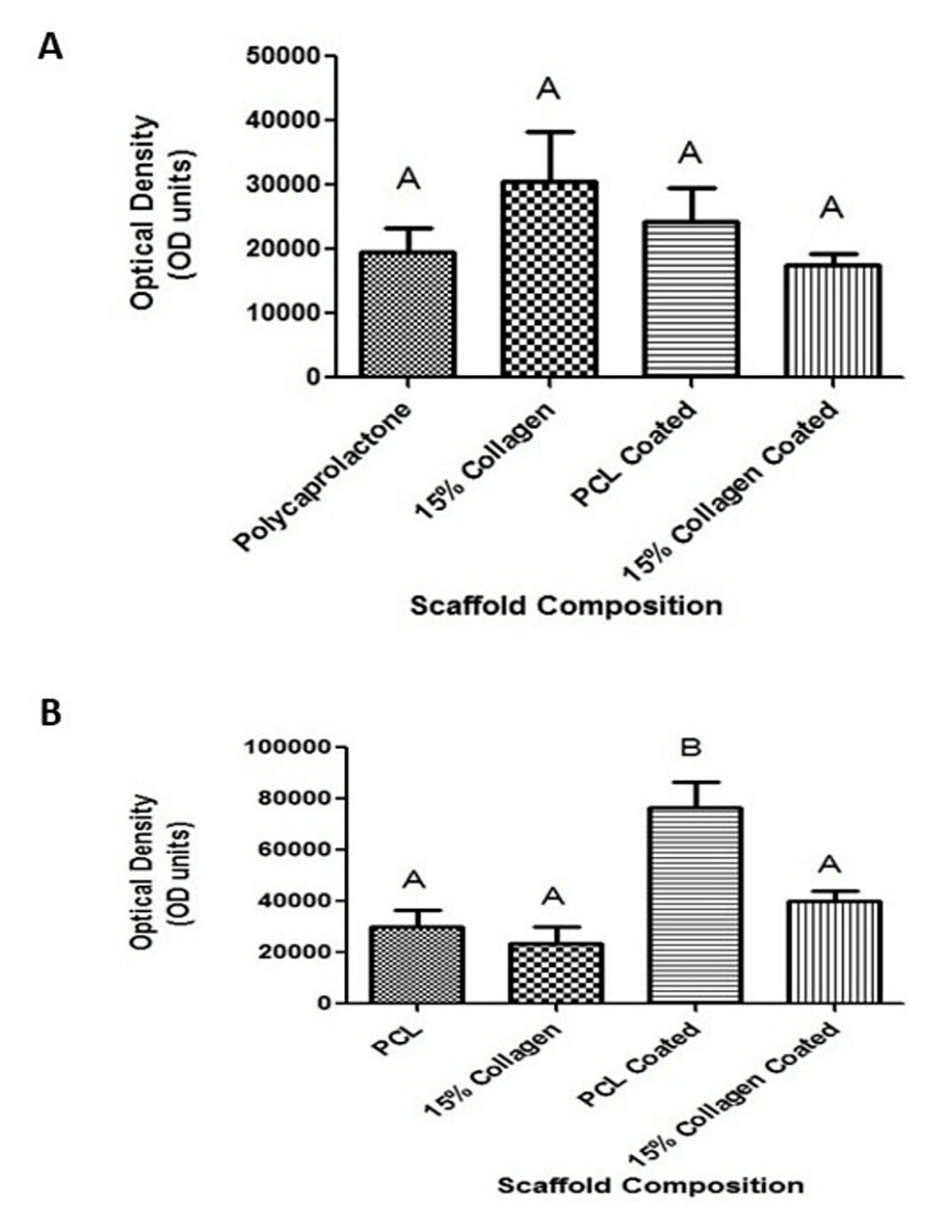
Scaffold collagen coating resulted in (A) no significant increases in initial cellular attachment and (B) an increase in proliferation for the polycaprolactone (PCL)-collagen-coated scaffold. Different letters above the error bars represent statistically significant differences between groups, similar letters were not significant, n = 3 per experimental group, and error bars represent the standard error of the mean

The effects of the scaffold on cellular attachment and proliferation were then examined and the results are plotted in Figure 11. These results indicate that the coating process did not significantly increase the initial adhesion of cells to the scaffold. However, it does indicate that the addition of collagen coating led to increased cellular proliferation for the PCL-coated material.

## Discussion

Many studies have indicated the advantages and nuances of utilizing electrospun scaffolds in regenerative medicine [5,9-12]. Furthermore, the inclusion of collagen in these scaffolds, through coating and as a part of the electrospinning process, has previously been investigated. However, discrepancies often arise in the reported mechanical and biocompatible properties, leading to rapid degradation. This may be due, in part, to the wide array of intended applications and scaffold preparation methodologies [13]. It has been previously shown that the method of surface treatment, solvent utilized, electrospinning parameters, and material concentrations all have a significant impact on the mechanical and biocompatible properties of these scaffolds [14]. Interestingly, the authors of this study have found little in the form of a thorough investigation of the optimization of each of these individual scaffold characteristics specific to tendons. As such, this study sought to thoroughly analyze how minute changes in concentration and the method of collagen inclusion could affect each aspect of the constructed scaffolds. Additionally, the study investigated how the modeling of these scaffolds could lead to consistent and highly reproducible macro-scale properties for clinical use in orthopedics.

In this regard, electrospinning with a protein such as collagen was found to cause significant mechanical and morphological changes. SEM imaging indicated ECM morphology with randomly aligned fibers. This particular morphology has been established as an ideal niche for stem cell proliferation [15,16]. Furthermore, this study demonstrated that fiber diameter decreases with collagen inclusion during the electrospinning process. This observation aligns with the scaffold attributes documented by Guzmán-Soria et al. who cite authors of supplementary investigations corroborating this congruent finding [17]. The scaffolds were also shown to be chemically altered with increasing amounts of collagen as shown through FTIR imaging. These results identified some bonding between collagen and PCL but did not provide any indication that collagen was fully integrated into the individual nanofibers of the scaffold. Furthermore, previous studies reported that collagen may be chemically altered in the process [18]. As such, further studies would provide insight into the exact molecular composition of the individual nanofibers in these scaffolds. AFM studies were able to quantify the mechanical properties of individual fibers [19]. The differences in mechanical properties observed in this study have the same trends as indicated in previous studies reported in the literature [20,21].

Following the completion of the physical and mechanical analyses of the scaffolds, each material was analyzed for its biocompatibility with autologous stem cells. Autologous stem cells have shown increasing promise in regenerative medicine [22-25]. However, these stem cells require a specified and highly characterized niche to ensure their successful proliferation and differentiation. Under indefinite conditions, pluripotent stem cells have shown the ability to spontaneously form teratomas [26]. Furthermore, stem cells have shown significant migration ability [27]. Without an accompanying scaffold to stabilize the microenvironment, these cells could migrate to distant locations of the body instead of the site of interest, potentially forming undesirable tissue foci. Scaffolds provide an environment for stem cell implantation and provide a viable device for surgical manipulation [5].

Additionally, the mechanical and morphological properties of the scaffold have a significant effect on the initial attachment and proliferation of autologous stem cells [28]. In this study, the introduction of collagen during the electrospinning process led to increased amounts of initial attachment by the stem cells. However, the coating of the scaffold surface with collagen leads to increased amounts of proliferation. This is similar to previous findings, which also noted that this finding may be due to a significant change in the hydrophobicity of the scaffolds [29]. Although not quantified in this study, it should be noted that a qualitative assessment of the scaffolds indicated a reduction in hydrophobicity as collagen was added. This was indicated during the hydration process, as the scaffolds with collagen included took significantly less time to absorb the media than the PCL-only scaffold. Furthermore, coating with collagen led to changes in mechanical strength but did not alter scaffold morphology in a significant manner. Additionally, the coatings of collagen lead to changes in the proliferation rates of adhered autologous stem cells. This is not surprising, as a collagen substrate would simulate more closely the ECM encountered in vivo.

The current literature explores electrospun PCL-collagen scaffolds for orthopedic applications with various efficacious compositions that address the limitations of conventional tendon repair techniques. Zhu et al. observed improved collagen organization and enthesis in rotator cuff tendon-bone healing through the addition of kartogenin, a cell differentiation promoter [30]. Aminatun et al. incorporated hydroxyapatite via electrospinning for an anterior cruciate ligament (ACL) scaffold. While achieving satisfactory live cell percentage and degradation rate, the study highlights the challenge of attaining mechanical strength equivalent to a native ACL [5]. Echoing biomechanical complexity, Gögele et al. emphasize the challenge of translating regenerative medicine research into practical applications, with increasing complexity raising difficulties and costs in obtaining medical device approval [31].

This study on electrospun scaffolds in regenerative medicine, particularly with collagen inclusion, presents valuable insights, but certain limitations merit consideration. Notably, the study lacks a comprehensive exploration of individual scaffold characteristics' optimization, hindering a nuanced understanding of minute changes. While the study validates collagen-induced mechanical and morphological alterations by SEM imaging and FTIR analysis, the study falls short in determining the exact molecular composition of the nanofibers. Highlighting the intricacies of scaffold design limitations, there is a call for future research to explore methodological variations in our scaffold and delve deeper into molecular intricacies.

## Conclusions

In summary, PCL and collagen scaffolds were electrospun at various concentrations. Our findings reveal that the inclusion of collagen during the electrospinning process results in increased ultimate tensile strength only at lower concentrations. Additionally, collagen inclusion within the scaffold altered stem cells' initial attachment and proliferation to its surface, which may be due to a change in its hydrophobicity. A collagen coating process of the scaffold did not alter the morphology but did result in changes in proliferation and tensile strength. As such, these various changes suggest that an ideal combination of electrospun collagen with coated collagen could be included for a PCL scaffold that would result in ideal mechanical strength, hydrophilicity, and surface properties for the proliferation of autologous stem cells. This study indicates that the optimization of each of these scaffold characteristics and subsequent finite element modeling can lead to highly repeatable and ideal scaffold properties for stem cell integration and proliferation. Future research should explore methodological variations and molecular intricacies of the PCL-collagen scaffold, aiming to enhance its efficacy in diverse orthopedic interventions, particularly in site-specific tendon repair and regeneration. In addition, preclinical studies followed by clinical studies are warranted to evaluate the safety and efficacy of PCL/collagen scaffold(s) for potential regenerative medicine applications, including tendon regeneration.
